# Using gut microbiome metagenomic hypervariable features for diabetes screening and typing through supervised machine learning

**DOI:** 10.1099/mgen.0.001365

**Published:** 2025-03-10

**Authors:** Xavier Chavarria, Hyun Seo Park, Singeun Oh, Dongjun Kang, Jun Ho Choi, Myungjun Kim, Yoon Hee Cho, Myung-hee Yi, Ju Yeong Kim

**Affiliations:** 1 Department of Tropical Medicine, Institute of Tropical Medicine, Arthropods of Medical Importance Resource Bank, Yonsei University College of Medicine, Yonsei-ro 50-1, Seodaemun-gu, Seoul 03722, Republic of Korea; 2 Department of Systems Biology, Yonsei University College of Life Science and Biotechnology, Yonsei-ro 50-1, Seodaemun-gu, Seoul 03722, Republic of Korea

**Keywords:** diabetes mellitus, gut microbiome, metabarcoding, microbial markers, random forest, supervised machine learning

## Abstract

Diabetes mellitus is a complex metabolic disorder and one of the fastest-growing global public health concerns. The gut microbiota is implicated in the pathophysiology of various diseases, including diabetes. This study utilized 16S rRNA metagenomic data from a volunteer citizen science initiative to investigate microbial markers associated with diabetes status (positive or negative) and type (type 1 or type 2 diabetes mellitus) using supervised machine learning (ML) models. The diversity of the microbiome varied according to diabetes status and type. Differential microbial signatures between diabetes types and negative group revealed an increased presence of *Brucellaceae*, *Ruminococcaceae*, *Clostridiaceae*, *Micrococcaceae*, *Barnesiellaceae* and *Fusobacteriaceae* in subjects with diabetes type 1, and *Veillonellaceae*, *Streptococcaceae* and the order *Gammaproteobacteria* in subjects with diabetes type 2. The decision tree, elastic net, random forest (RF) and support vector machine with radial kernel ML algorithms were trained to screen and type diabetes based on microbial profiles of 76 subjects with type 1 diabetes, 366 subjects with type 2 diabetes and 250 subjects without diabetes. Using the 1000 most variable features, tree-based models were the highest-performing algorithms. The RF screening models achieved the best performance, with an average area under the receiver operating characteristic curve (AUC) of 0.76, although all models lacked sensitivity. Reducing the dataset to 500 features produced an AUC of 0.77 with sensitivity increasing by 74% from 0.46 to 0.80. Model performance improved for the classification of negative-status and type 2 diabetes. Diabetes type models performed best with 500 features, but the metric performed poorly across all model iterations. ML has the potential to facilitate early diagnosis of diabetes based on microbial profiles of the gut microbiome.

**Impact Statement**

Diabetes is a major public health concern with a rapidly increasing prevalence and the potential to have a significant impact on healthcare systems and quality of life. Our study explores the application of supervised machine learning for diabetes detection and typing based on hypervariable sOTU features of the gut microbiome from a public dataset of a volunteer citizen science initiative. Machine learning approaches such as these open the door to their application for early prediction of diabetes, with the goal of identifying at-risk individuals before disease onset. This research contributes to the field by demonstrating how gut microbiome sOTU profiles, combined with machine learning, could support proactive management and prevention of diabetes, offering the possibility of early intervention, personalized healthcare and enabling lifestyle changes.

## Data Summary

Four supplementary figures and four supplementary tables are available with the online version of this article. All sequencing data used in the present study were obtained from the Qiita study ID 10317. Sample QIITA IDs are presented in Table S1. R scripts and Qiime2 code are deposited in the GitHub repository xcbayot/diabetesml.

## Introduction

Diabetes is a multifactorial and polygenic group of metabolic and immune diseases characterized by chronic hyperglycaemia, insulin deficiency and impaired glucose metabolism [1]. These diseases pose a major threat to public health in both developed and developing countries due to their rapid growth and increase in prevalence in young people worldwide over the past decades [2]. Global prevalence estimates from the 10th edition of the IDF Diabetes Atlas exceeded half a billion people aged 20–79 years and are projected to reach 800 million by 2045 [3]. Diabetes has become one of the most common chronic diseases, making its management and early detection a priority to prevent serious health complications and increased healthcare expenditures [4].

Type 1 diabetes mellitus (T1DM) is characterized by the autoimmune destruction of *β*-cells within the pancreatic islets of Langerhans, resulting in insulin deficiency [5]. Type 2 diabetes mellitus (T2DM) involves peripheral multi-organ insulin resistance, pancreatic *β*-cell dysfunction and chronic inflammation in the pancreas [6]. While both diseases are phenotypically and aetiologically heterogeneous, multiple factors such as genetic predisposition, sedentary lifestyle, dietary patterns, oxidative stress and obesity are known to play roles in their pathogenesis [2]. Diabetes is also associated with various comorbidities such as thyroid disorders and cardiovascular diseases [7]. In particular, epilepsy and cardiovascular diseases have been reported as a comorbidity of T1DM [8,10]. T2DM is often associated with adipose tissue infiltration, atherosclerosis and amyloid plaque deposition in the pancreas, as well as anxiety and depression [11,12].

Recent research has increasingly highlighted the crucial role of the gut microbiota in the pathophysiology of many multifactorial diseases, including diabetes [13]. The interactions between the host body and the microbiome have an important effect on glucose metabolism, insulin resistance and inflammation [14]. Dysbiosis, an imbalance in the normal microbial community of the host, either results from or contributes to the development and progression of T1DM and T2DM and is implicated in metabolic dysfunction [15]. The gut microbiome composition of healthy individuals differs significantly in both functional and taxonomic aspects from that of patients with diabetes, obesity and other metabolic diseases [16]. Microbiota transfers from lean human donors into recipients with metabolic syndrome are known to reduce insulin resistance in the recipient [17], and germ-free mice that receive microbiota transplants from either obese or lean human twins adopt the donor phenotype [18]. Interestingly, microbiota from lean human donors can decrease fat accumulation in obese recipient mice, and cohousing of obese and lean mice recapitulates a lean-like phenotype and microbiota profile in obese mice [18].

There is broad consensus that these microbial interactions can be exploited for therapeutic strategies aimed at modulating the gut microbiome to manage and prevent diseases, as well as to discover disease biomarkers that can be used as a predictive approach before their onset [19]. However, multifactorial diseases are rarely directly related to a single set of microbes but rather can be influenced by a variety of microbial interactions changing according to niche colonization, abiotic factors and host biology [20]. To this end, computational-based models like machine learning (ML) algorithms, deep learning and artificial intelligence have been consistently used to analyse extensive metagenomics datasets and trained to classify different multifactorial diseases as they incorporate the variability of the gut microbiome [21,23]. These approaches will take advantage of the constantly increasing size of the available human microbiome data and will provide an alternative to traditional statistical models by accounting for the complex variations of the gut microbiome among subjects. Here, we trained a set of supervised ML algorithms with 16S rRNA gut metagenomic data from a group of human volunteers from the American Gut Project diagnosed with T1DM and T2DM to screen for microbial markers associated with each of the two diabetes types [24]. Given the growing evidence of the link between the gut microbiota and the pathogenesis of diabetes, we hypothesize that these biomarker-based supervised ML models will be able to detect and type diabetes based on the gut microbial profiles of the American Gut Project volunteers. While diabetes and its types are easily diagnosed through biochemical tests, ML approaches have the potential to be used to predict the onset of diabetes based on gut microbiome profiles. By identifying bacterial profiles of patients who have developed diabetes, future models could identify individuals at risk of developing diabetes, allowing for early intervention, personalized healthcare strategies and lifestyle changes.

## Methods

### Data collection

Metagenomic data (16S rRNA) from the American Gut Project (Qiita study ID 10317) [24] were obtained with the Redbiom software [25] from the Qiita platform [26] using the context Deblur_2021.09-Illumina-16S-V4-150nt-ac8c0b (Fig. S1, available in the online Supplementary Material). A total of 76 samples of subjects with T1DM, 366 samples from subjects with T2DM and 250 samples from subjects without any diabetes type (healthy individuals) were obtained. Inclusion parameters for subjects with diabetes were subjects diagnosed with diabetes by a medical professional [diabetes=diagnosed by a medical professional (doctor, physician assistant)] and those whose diabetes type was either T1DM or T2DM (diabetes_type=‘type 1 diabetes’ OR ‘type 2 diabetes’) not suffering from inflammatory bowel disease (IBD) (ibd=‘I do not have this condition’). Exclusion parameters were subjects with IBD [ibd=‘diagnosed by a medical professional (doctor, physician assistant)’ OR ‘self-diagnosed’ OR ‘not provided’ OR ‘diagnosed by an alternative medicine practitioner’]. Exclusion parameters for subjects considered without diabetes were those diagnosed with any other type of diabetes [diabetes=‘diagnosed by a medical professional (doctor, physician assistant)’ OR ‘self-diagnosed’ OR ‘not provided’ OR ‘diagnosed by an alternative medicine practitioner’] or IBD [ibd=‘diagnosed by a medical professional (doctor, physician assistant)’ OR ‘self-diagnosed’ OR ‘not provided’ OR ‘diagnosed by an alternative medicine practitioner’]. After applying the exclusions, a random shuffle of the filtered data was performed using the shuf command to ensure variability in the sample datasets, and the corresponding biom and metadata files were then fetched for analysis. The sequences were imported into QIIME2, and the representative sequences were extracted and merged [27].

### Taxonomic assignment and marker identification

The sub-operational taxonomic units (sOTUs) table generated by the Deblur pipeline was used for taxonomic identification [28]. Taxonomic identity of the sOTU features was assigned with the Naïve Bayes classifier trained on the Greengenes v. 13.8 OTU reference tree clustered at 99% sequence similarity [29]. The classification process was carried out in QIIME2 using the feature-classifier classify-sklearn plugin [30]. Linear discriminant analysis (LDA) effect size (LEfSe) [31] was performed in the R software v.4.3.3 (https://www.R-project.org/) using the microeco package [32] to characterize the representative bacterial taxa from the disease status groups (positive-status and negative-status) as well as for the two diabetes types (T1DM or T2DM). Taxa were considered enriched in a group if they met the criteria of an FDR-adjusted *P*-value<0.05 and an LDA score≥2.0. The analysis was conducted on a rarefied dataset standardized to 1000 reads per sample. Other default LEfSe parameters were maintained. The top 50 most representative taxa, identified across all taxonomic levels by LEfSe, were visualized on a cladogram, which was constructed from the top 1000 most abundant taxa in the microbiome. The cladogram highlights enriched taxa and their phylogenetic relationships.

### Diversity metrics comparisons

The sOTUs were converted into a microtable object with the phyloseq and microeco packages in the R software v.4.3.3 [33]. Diversity metrics of the gut microbiome were calculated with the microeco package using the trans_alpha and trans_beta functions after rarefaction to a depth of 1000 reads to ensure that low read samples were not carried to the subsequent analyses but retained variation in the dataset. Shannon index and observed features number were calculated for alpha diversity metrics, and unweighted UniFrac distances were calculated for beta diversity metrics and plotted using principal coordinate analysis (PCoA). Alpha diversity metrics were compared with the Wilcoxon rank-sum test, and beta diversity metrics were compared using permutational multivariate analysis of variance (PERMANOVA) with the microeco package to test diversity differences between diabetes types and disease status.

### ML algorithms training stage

The bacterial sOTUs from the phyloseq were normalized using R. The normalized bacterial sOTUs dataset was used as the variable input to train the decision tree (DT) and elastic net (EN) interpretable ML models, and the random forest (RF) and support vector machine with radial kernel (SVMRK) black-box models followed the strategy published by Aryal *et al*. [34]. All models were trained using the caret package [35] in the R software. For the training step, 70% of the dataset was randomly selected and the top 1000 sOTU features with the highest variance across the subset were used to reduce underfitting, as sOTUs with higher variance are more likely to be more informative. Repeated *k*-fold cross-validation (five repeats of fivefold cross-validation) at the training stage was performed for training performance evaluation with the repeated option. Hyperparameter tuning was performed by evaluating ten different values for each hyperparameter. This process was repeated to train the model to classify positive-status vs. negative-status and T1DM vs. T2DM. Since T2DM is closely related to lifestyle factors and age [36], we repeated the models to classify negative-status vs. positive-status while only including the T2DM subjects.

The training was then performed on a reduced 500 and 100 hypervariable features dataset in order to increase sensitivity. In addition to this analysis, another round of models was run using log10 normalization of relative abundances to reduce the importance of outliers and make the relationships within the data more interpretable to the models. To accommodate the varying complexity, the iteration maxits of the EN and SVMRK models were set to 10 000 iterations. The maximum depth of the DT model was set to 30 to account for the complexity of the metagenomic data and to avoid excessive overfitting. The number of trees of the RF analyses was set to 1000 to ensure model stability and to capture intricate relationships in the sOTU data as it is less prone to overfitting. To address the low sensitivity and high specificity of the models, suggesting a conservative threshold for recall biased towards negative classification, the classification threshold of the predicted probabilities was changed from 0.5 to 0.60, 0.65 and 0.70 and then reduced to 0.30, 0.35 and 0.40 using the prob_predictions function, which converts the continuous probability scores of the models into discrete classes, in order to explore the trade-offs between true positives and false positives.

### ML algorithms testing stage

Models were tested on the remaining 30% of the dataset, and performance was evaluated using the following validation metrics: area under the receiver operating characteristic curve (AUC), sensitivity, specificity, accuracy, positive predictive value (PPV, a measure of precision) and F1 score. A total of 50 independent iterations of microbiome-based supervised ML training and testing were repeated and performance metrics for each model were compiled into a data frame. The dataset was shuffled and split into training and testing sets before each iteration to ensure different group compositions in every iteration. The performance metrics of the models trained with 1000, 500 and 100 features were compared using Kruskal–Wallis tests, with post-hoc pairwise Wilcoxon rank-sum tests using Holm correction with the stats package in R. Finally, the variable importance scores belonging to each sOTU for each model across all iterations were aggregated to define the most influential highly contributing features for both screening and typing. The scripts for these analyses were deposited in the GitHub repository xcbayot/diabetesml.

## Results

### Diversity metrics comparison

**Fig. 1.**
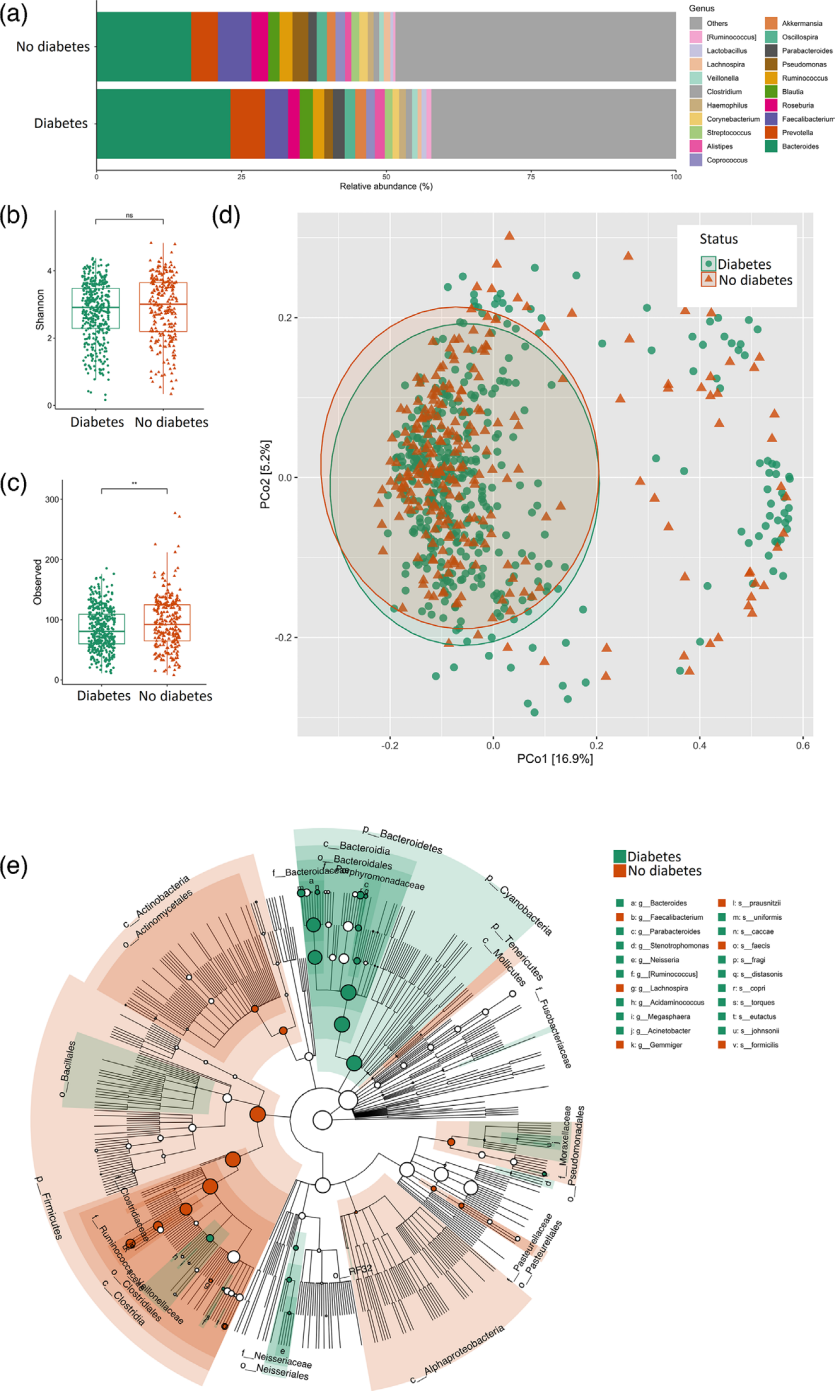
Diversity metrics of the gut microbiome from volunteers that reported diabetes vs. volunteers that did not report diabetes. Mean relative abundance of the top 25 gut microbiome sOTUs of volunteers that reported diabetes vs. volunteers that did not report diabetes at the genus level (**a**). Shannon index of the gut microbiome sOTUs of volunteers that reported diabetes vs. volunteers that did not report diabetes compared by Wilcoxon rank-sum test (**b**). Richness of the gut microbiome sOTUs of volunteers that reported diabetes vs. volunteers that did not report diabetes compared by Wilcoxon rank-sum test (**c**). Unweighted UniFrac distances PCoA of the gut microbiome of volunteers that reported diabetes vs. volunteers that did not report diabetes tested with PERMANOVA (**d**). Cladogram of the top 50 more representative taxa based on LEfSe analysis from the gut microbiome of volunteers that reported diabetes vs. volunteers that did not report diabetes showing their phylogenetic relationship with the top 1000 most abundant taxa, labels in the cladogram show sOTUs (LDA>2, *P*<0.5) of superior taxonomic levels, labels outside the cladogram represent enriched sOTUs at the genus and species levels (**e**).

The most abundant taxa from the diabetes positive-status and negative-status groups were *Bacteroides*, *Prevotella*, *Faevalibacterium*, *Roseburia*, *Blautia*, *Ruminococcus*, *Pseudomonas*, *Parabacteroides*, *Oscillospira* and *Akkermansia* (Fig. 1a). Shannon index did not vary significantly between both groups (*P*=0.160, Fig. 1b). However, richness was higher in the negative-status group (*P*=0.003, Fig. 1c). Beta diversity (unweighted UniFrac distances) differed between both groups (*P*=0.006, Fig. 1d), and a total of 22 biomarkers were found for both conditions at the genus and species levels (Fig. 1e). Among the significantly enriched sOTUs from the diabetes positive group were the following: *Acinetobacter johnsonii*, *Coprococcus eutactus*, *Ruminococcus torques*, *Prevotella copri*, *Parabacteroides distasonis*, *Pseudomonas fragi*, *Bacteroides caccae*, *Bacteroides uniformis*, *Stenotrophomonas*, *Neisseria*, *Acidaminococcus* and *Megasphaera*. At superior taxonomic levels, the phylum *Bacteroidetes*, the order *Bacillales* and the families *Fusobacteraceae*, *Moraxellaceae*, *Neisseriaceae* and *Veillonellaceae* were also microbial markers of this condition. The non-diabetes group presented *Gemmiger formicilis*, *Roseburia faecis*, *Faecalibacterium prausnitzii* and *Lachnospira*. Enriched superior taxonomic levels were the phyla *Firmicutes* and *Tenericutes*, the class *Alphaproteobacteria*, the orders *Pseudomonadales* and *Actinomycetales* and the families *Moraxellaceae*, *Pasteurellaceae*, *Clostridiaceae* and *Ruminococcaceae*. Additionally, when comparing negative-status subjects with T2DM subjects, alpha diversity varied significantly (Fig. S2). The negative-status group had a higher Shannon index (*P*=0.028) and feature richness (*P*<0.001). Beta diversity also showed a significant difference (*P*=0.01).

**Fig. 2.**
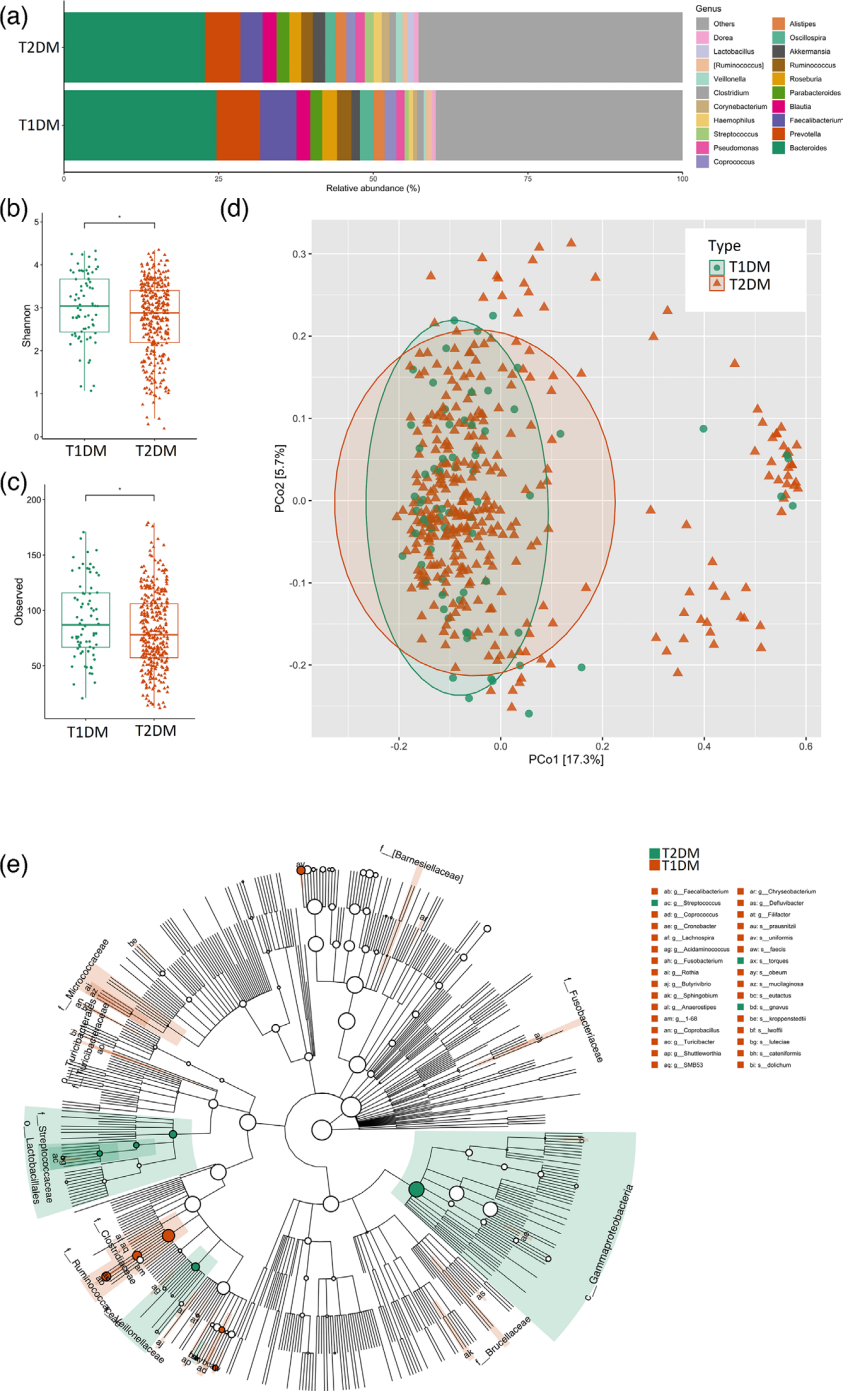
Diversity metrics of the gut microbiome from volunteers that reported diabetes type 1 (T1DM) vs. volunteers that reported diabetes type 2 (T2DM). Mean relative abundance of the top 25 gut microbiome sOTUs of volunteers that reported diabetes type 1 vs. volunteers that reported diabetes type 2 at the genus level (**a**). Shannon index of the gut microbiome sOTUs of volunteers that reported diabetes type 1 vs. volunteers that reported diabetes type 2 compared by Wilcoxon rank-sum test (**b**). Richness of the gut microbiome sOTUs of volunteers that reported diabetes type 1 vs. volunteers that reported diabetes type 2 compared by Wilcoxon rank-sum test (**c**). Unweighted UniFrac distances PCoA of the gut microbiome of volunteers that reported diabetes type 1 vs. volunteers that reported diabetes type 2 tested with PERMANOVA (**d**). Cladogram of the top 50 more representative taxa based on LEfSe analysis from the gut microbiome of volunteers that reported diabetes type 1 vs. volunteers that reported diabetes type 2 showing their phylogenetic relationship with the top 1000 most abundant taxa, labels in the cladogram show sOTUs (LDA>2, *P*<0.5) of superior taxonomic levels, labels outside the cladogram represent enriched sOTUs at the genus and species levels (**e**).

Overall, the distribution of the most abundant taxa was similar for the partition of T1DM and T2DM (Fig. 2a). Both groups varied in Shannon index (*P*=0.046, Fig. 2b) and richness (*P*=0.047, Fig. 2c). Beta diversity (unweighted UniFrac distances) was different between both groups (*P*=0.001, Fig. 2d), and a total of 32 biomarkers were found for both conditions at the genus and species levels (Fig. 2e). The enriched taxa from the gut microbiome of T1DM subjects at the genus and species levels were *F. prausnitzii*, *B. uniformis*, *R. faecis*, *Blautia obeum*, *Rothia mucilaginosa*, *C. eutactus*, *Corynebacterium kroppenstedtii*, *Acinetobacter lwoffii*, *Streptococcus luteciae*, *Coprobacillus cateniformis*, *Eubacterium dolichum*, *Butyrivibrio*, *Cronobacter*, *Lachnospira*, *Sphingobium*, *Anaerostipes*, *Coprobacillus*, *Turicibacter*, *Shuttleworthia*, *Chryseobacterium*, *Defluvibacter* and *Filifactor*. Among superior taxonomic levels enriched in this condition were the families *Brucellaceae*, *Ruminococcaceae*, *Clostridiaceae*, *Micrococcaceae*, *Barnesiellaceae* and *Fusobacteriaceae*. The enriched taxa of the T2DM group were *R. torques*, *Ruminococcus gnavus*, *Streptococcus*, the families *Veillonellaceae* and *Streptococcaceae* and the order *Gammaproteobacteria*.

### ML models performance

The DT, EN, RF and SVMRK models were trained with 70% of the dataset using the top 1000 highest-variance sOTUs from the gut microbiome. Then AUC, sensitivity, specificity, accuracy, precision and F1 score were calculated across 50 independent iterations of ML testing (Fig. S3).

Overall, RF (AUC=0.76±0.03) and DT (AUC=0.64±0.05) performed best for detecting diabetes status. Sensitivity and PPV were low for both RF (sensitivity=0.46±0.06, PPV=0.64±0.06) and DT (sensitivity=0.47±0.1, PPV=0.52±0.06) models. EN and SVMRK models performed poorly despite displaying similar AUC values. Furthermore, models failed to classify diabetes type (Table S2).

**Fig. 3.**
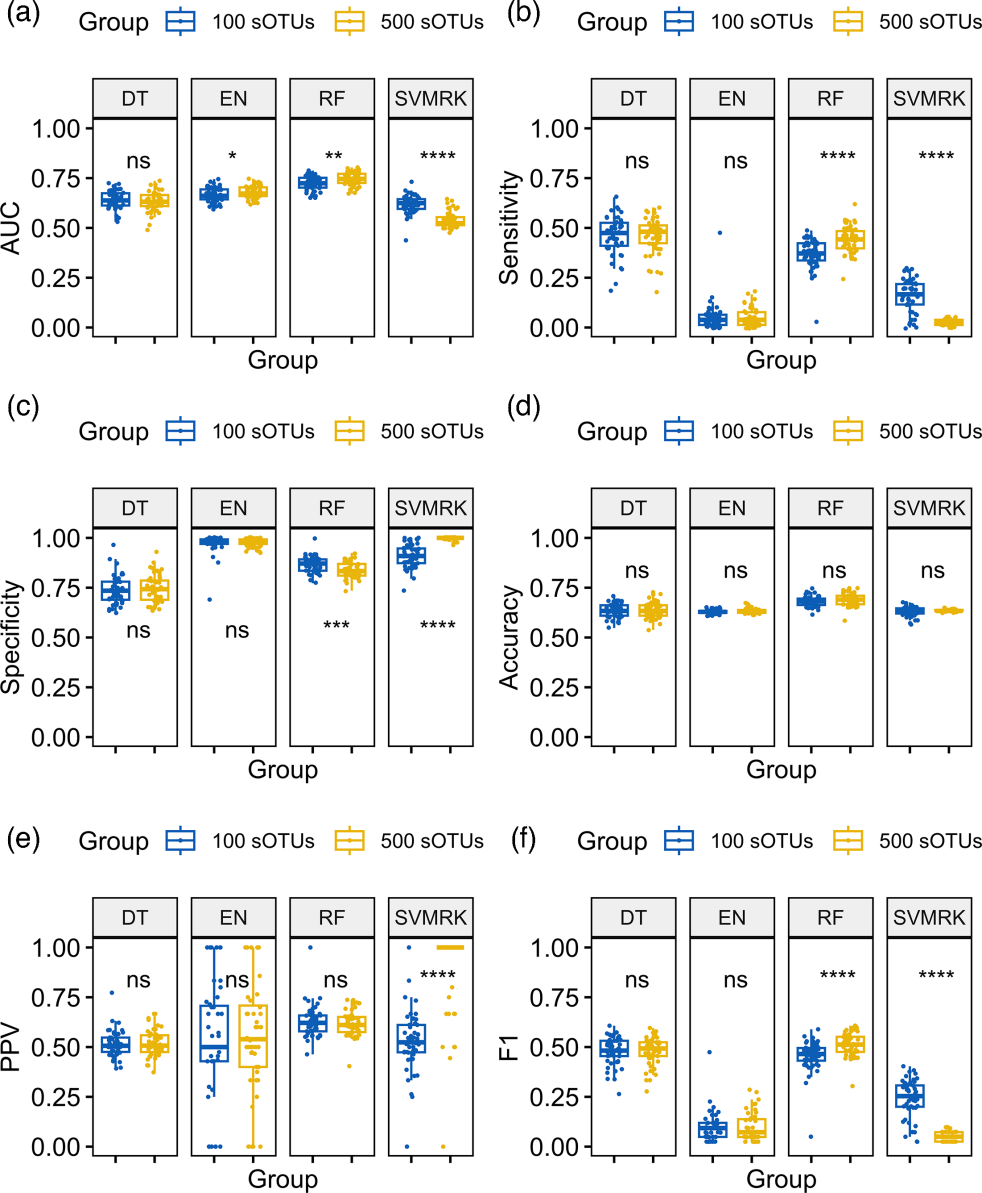
Performance metrics of four supervised ML algorithms for the screening of diabetes status trained on the top 100 and 500 hypervariable sOTUs of the gut microbiome across 50 iterations. Diabetes status was screened with the DT, EN, RF and SVMRK models. AUC (**a**), sensitivity (**b**), specificity (**c**), accuracy (**d**), PPV (**e**), F1 score (**f**). The models use a predicted probability classification threshold of 0.5 with default iterations and tree maxit levels.

**Fig. 4.**
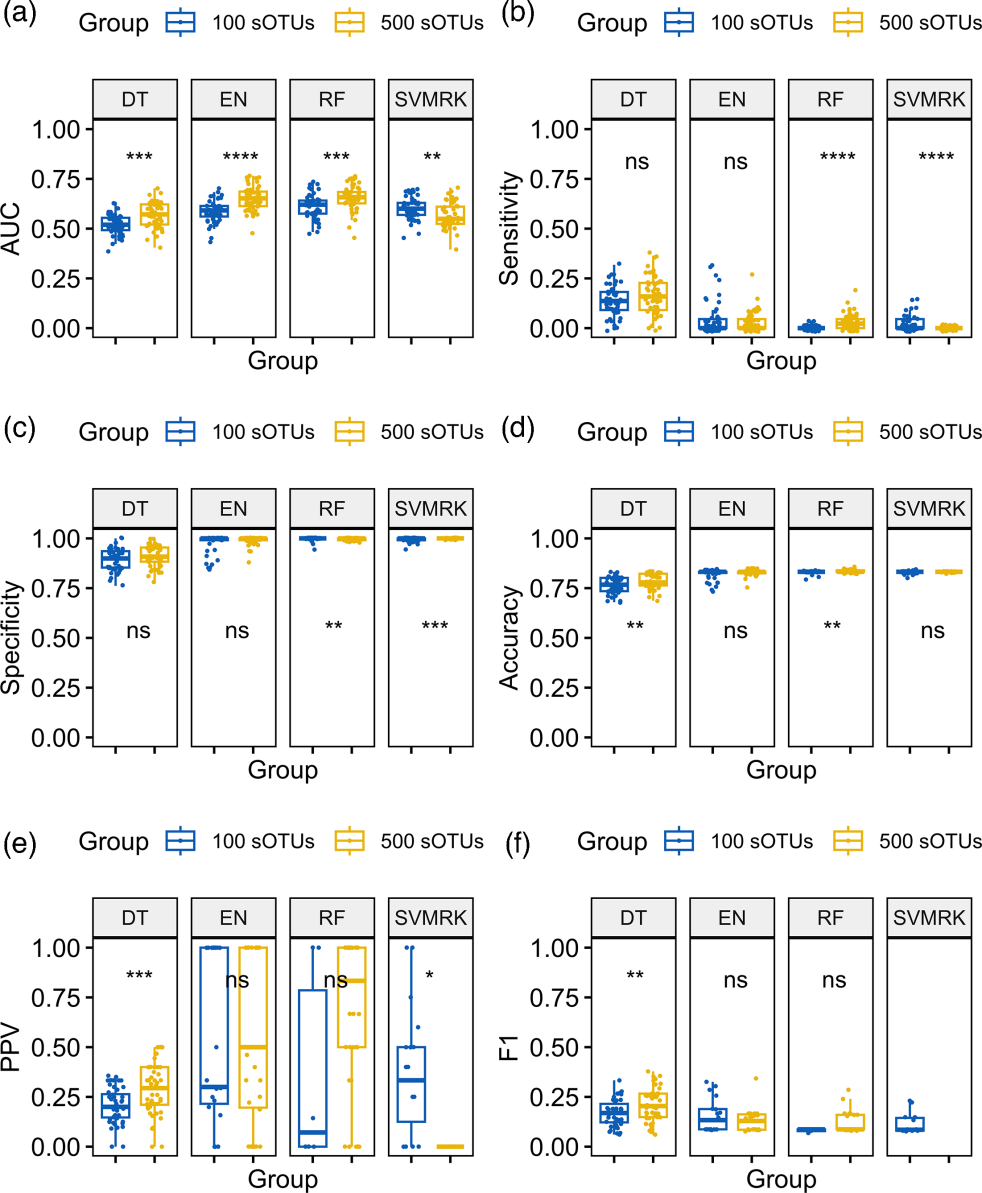
Performance metrics of four supervised ML algorithms for the typing of diabetes trained on the top 100 and 500 hypervariable sOTUs of the gut microbiome across 50 iterations. Diabetes status was screened with the DT, EN, RF and SVMRK models. AUC (**a**), sensitivity (**b**), specificity (**c**), accuracy (**d**), PPV (**e**), F1 score (**f**). The models use a predicted probability classification threshold of 0.5 with default iterations and tree maxit levels.

Models were retrained with the top 500 and 100 sOTUs for both diabetes status (Fig. 3) and type (Fig. 4) classification. Overall, RF achieved the highest AUC for diabetes status in the 500 features dataset (AUC=0.75±0.005), significantly higher (*P*=0.003) than the 100 features dataset (AUC=0.72±0.005). Sensitivity was the highest in the DT models (100 sOTUs sensitivity=0.46±0.014, 500 sOTUs sensitivity=0.46±0.013, *P*=0.899), followed by the RF, which performed better with 500 features (0.44±0.009, *P*<0.001). Specificity was the same in both datasets in the DT models (0.74±0.01, *P*=0.742) and significantly higher in RF for the 100 features dataset (specificity=0.87±0.006, *P*<0.001). Detailed comparisons are provided in Table 1.

**Table 1.** Performance metrics comparisons of DT, EN, RF and SVMRK supervised ML algorithms for the screening and typing of diabetes trained on the top 100 and 500 hypervariable sOTUs of the gut microbiome across 50 iterations

Group	Classification	Model	AUC	*P*-value	Sensitivity	*P*-value	Specificity	*P*-value	Accuracy	*P*-value	PPV	*P*-value	F1	*P*-value
100 sOTUs	Diabetes status	DT	0.64±0.006	0.379	0.46±0.014	0.899	0.74±0.010	0.742	0.64±0.005	0.949	0.52±0.009	0.839	0.48±0.009	0.817
500 sOTUs	Diabetes status	DT	0.63±0.007		0.46±0.013		0.74±0.010		0.64±0.006		0.52±0.009		0.48±0.009	
100 sOTUs	Diabetes status	EN	0.66±0.005	0.037	0.05±0.010	0.629	0.97±0.007	0.682	0.63±0.002	0.449	0.62±0.034	0.648	0.10±0.013	0.873
500 sOTUs	Diabetes status	EN	0.68±0.004		0.05±0.010		0.98±0.003		0.63±0.002		0.62±0.035		0.10±0.011	
100 sOTUs	Diabetes status	RF	0.72±0.005	0.003	0.37±0.011	<0.001	0.87±0.006	<0.001	0.68±0.004	0.066	0.63±0.011	0.686	0.46±0.011	<0.001
500 sOTUs	Diabetes status	RF	0.75±0.005		0.44±0.009		0.84±0.006		0.69±0.004		0.62±0.009		0.51±0.008	
100 sOTUs	Diabetes status	SVMRK	0.62±0.006	<0.001	0.16±0.117	<0.001	0.91±0.008	<0.001	0.63±0.003	0.391	0.54±0.019	<0.001	0.24±0.014	<0.001
500 sOTUs	Diabetes status	SVMRK	0.54±0.006		0.02±0.002		0.99±0.001		0.64±0.001		0.92±0.025		0.05±0.003	
100 sOTUs	Diabetes type	DT	0.52±0.007	<0.001	0.15±0.010	0.0984	0.88±0.008	0.051	0.76±0.006	0.003	0.21±0.011	<0.001	0.17±0.009	0.009
500 sOTUs	Diabetes type	DT	0.57±0.009		0.18±0.013		0.90±0.007		0.78±0.006		0.30±0.016		0.21±0.012	
100 sOTUs	Diabetes type	EN	0.61±0.013	<0.001	0.12±0.022	0.284	0.95±0.014	0.306	0.81±0.009	0.156	0.62±0.085	0.514	0.16±0.019	0.181
500 sOTUs	Diabetes type	EN	0.66±0.011		0.08±0.010		0.98±0.006		0.83±0.004		0.66±0.066		0.13±0.123	
100 sOTUs	Diabetes type	RF	0.60±0.019	0.089	0.05±0.00	0.281	0.98±0.018	0.865	0.82±0.015	0.417	0.71±0.286	0.865	0.08±0.006	0.272
500 sOTUs	Diabetes type	RF	0.64±0.012		0.07±0.007		0.99±0.001		0.84±0.001		0.81±0.051		0.118±0.011	
100 sOTUs	Diabetes type	SVMRK	–	–	–	–	–	–	–	–	–	–	–	–
500 sOTUs	Diabetes type	SVMRK	–	–	–	–	–	–	–	–	–	–	–	–

For diabetes type classification, EN (0.66±0.011) and RF (0.64±0.012) performed best when trained on the 500 features dataset, but sensitivity was consistently low. Specificity was overall high across all models, reflecting a low false positive rate, meaning that non-T2DM samples were correctly classified as negative. However, sensitivity was low across all models, indicating that T2DM samples were not being correctly identified.

**Fig. 5.**
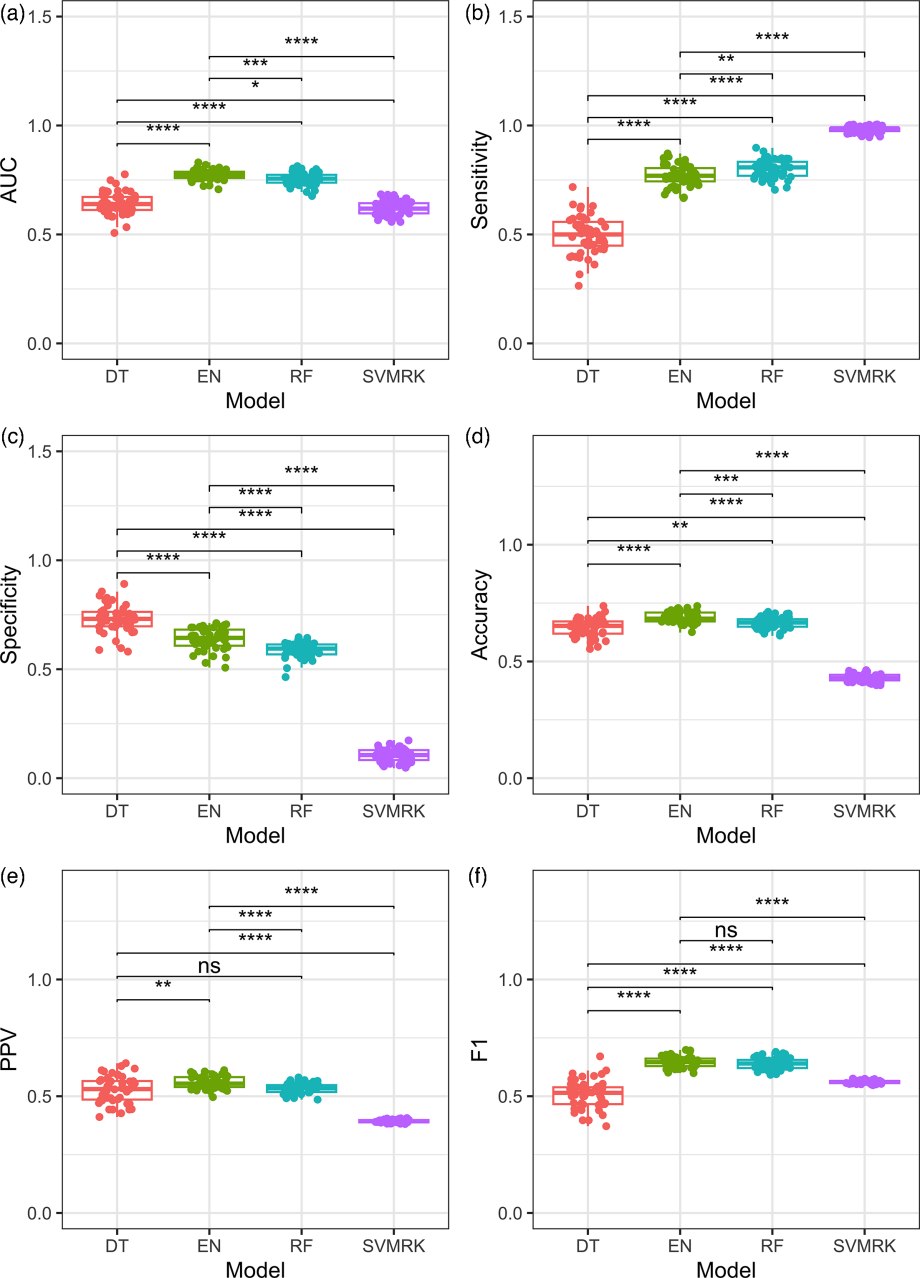
Performance metrics of four supervised ML algorithms for the screening of diabetes trained on the top 500 hypervariable sOTUs of the gut microbiome across 50 iterations. AUC (**a**), sensitivity (**b**), specificity (**c**), accuracy (**d**), PPV (**e**), F1 score (**f**). The models use a predicted probability classification threshold of 0.65 with 10000 maximum iterations for the EN and SVMRK models, maximal depth of 30 for the DT, 1000 maximum trees for the RF models.

To address low sensitivity, reflecting a low true positive rate and a high number of false negative classifications, the training process was repeated using the top 500 features with adjusted probability prediction thresholds and increased iterations. A threshold of 0.65 resulted in increased sensitivity values for diabetes status screening (Fig. 5). While AUC values generally remained unchanged in this version of the models, EN and RF were the best-performing algorithms (*P*<0.001), with EN achieving the highest AUC (0.77±0.02, *P=*0.002). While AUC for the RF model was slightly lower (0.75±0.03), RF displayed the highest sensitivity (0.80±0.04, *P*<0.001), followed by EN (0.77±0.05, *P*=0.002). DT had the highest specificity (0.73±0.06, *P*<0.001), followed by EN (0.64±0.05) and RF (0.59±0.03).

When focusing on T2DM vs. non-diabetic sample subjects (Fig. S4), EN and RF performed the best (Table 2). EN outperformed RF (AUC=0.82±0.027 vs. 0.80±0.029, *P*<0.001), with no significant difference in sensitivity (EN=0.85±0.052, RF=0.87±0.039, *P=*0.15), but higher specificity in EN (0.63±0.053 vs. 0.57±0.046, *P*<0.001). Furthermore, AUC was significantly higher (*P*<0.001) when training RF diabetes screening models using only the T2DM positive subjects rather than both T1DM and T2DM subjects. The same was seen for the sensitivity (*P*<0.001) and specificity metrics (*P*=0.009). The EN models showed the same differences in AUC (*P*<0.001) and sensitivity (*P*<0.001), but no differences in specificity were found (*P*=0.250). Table S3 summarizes these results.

**Table 2.** Comparison of performance metrics between ML models for the classification of negative diabetes status vs. positive-status and negative-status vs. T2DM subjects The RF and EN model metrics trained on the top 500 high-variance sOTUs are shown.

Classification	Model	AUC	Sensitivity	Specificity	PPV	Accuracy	F1
Status (Negative vs. Positive)	EN	0.77±0.024	0.77±0.047	0.64±0.048	0.56±0.026	0.69±0.025	0.65±0.024
Status (Negative vs. T2DM)	EN	0.82±0.027	0.85±0.052	0.63±0.053	0.62±0.003	0.72±0.027	0.72±0.026
Status (Negative vs. Positive)	RF	0.75±0.028	0.80±0.043	0.59±0.034	0.53±0.021	0.67±0.024	0.64±0.024
Status (Negative vs. T2DM)	RF	0.80±0.029	0.87±0.039	0.57±0.046	0.59±0.025	0.69±0.026	0.70±0.023

### Contributing features in ML models

Among the most influential sOTUs in the EN model for diabetes status screening (negative-status vs. positive-status of both types) were the families *Clostridiaceae*, *Planococcaceae*, *Ruminococcaceae*, *Lachnospiraceae*, *Erysipelotrichaceae*, the genera *Acidaminococcus* of the family *Veillonellaceae*, *Neisseria* (*Neisseriaceae*), *Dorea* (*Lachnospiraceae*), *Clostridium*, *Oscillospira* (*Ruminococcaceae*), *Shuttleworthia* (*Lachnospiraceae*), *Blautia* (*Lachnospiraceae*) and the species *P. copri* (*Prevotellaceae*), *Clostridium aldenense* (*Clostridiaceae*), *F. prausnitzii* (*Ruminococcaceae*) and *B. caccae* (*Bacteroidaceae*). The most important features in the DT model were the order *Clostridiales*, the families *Clostridiaceae* and *Ruminococcaceae*, the genus *Clostridium* and the species *F. prausnitzii*. Finally, the most important features in the RF model were the order *Clostridiales*, the families *Clostridiaceae*, *Ruminococcaceae*, *Lachnospiraceae*, *Planococcaceae*, *Erysipelotrichaceae* and *Enterobacteriaceae*, the genera *Neisseria*, *Clostridium*, *Phocaeicola* (*Bacteroidaceae*), *Pseudomonas* (*Pseudomonadaceae*), *Lactococcus* (*Streptococcaceae*) and the species *F. prausnitzii*, *P. copri*, *R. torques*, *Bacteroides thetaiotaomicron*, *B. uniformis*, *P. fragi* and *Haemophilus parainfluenzae*. Among the contributing features in the EN models classifying negative-status vs. T2DM were *Prevotella*, *Planococcaceae*, *Lactobacillus*, *Clostridiaceae*, *Shuttleworthia*, *Acidaminococcus*, *Oscillospira*, *R. mucilaginosa* and *Ruminococcus bromii*. The features *F. prausnitzii*, *Erysipelotrichaceae*, *Clostridiaceae*, *Lachnospiraceae*, *R. mucilaginosa*, *P. copri*, *Bacteroides* and *P. distasonis* were important for the DT models classification. Finally, *Clostridiaceae*, *F. prausnitzii*, *Erysipelotrichaceae*, *Lachnospiraceae*, *R. torques* and *P. copri* were important features of the RF models. The aggregated importance scores for the top 500 most variable sOTU features for the diabetes status models for the negative vs. positive and the negative vs. T2DM classifications are shown in Table S4.

## Discussion

While diabetes can be easily diagnosed using biochemical strategies, the increase in diabetes prevalence over the last few decades makes it urgent to explore approaches aimed at early prediction of the disease [37]. As taxonomic and functional differences in the gut microbiome are characteristic of diabetic patients [38], the gut microbiome profiles, combined with ML, could predict individuals at risk of developing diabetes, which in turn could help to contribute to the knowledge of the poorly understood relationship between the gut microbiome and diabetes. ML strategies have been widely used to exploit patient-derived data in order to predict multifactorial diseases [39,40]. In the present study, we applied a series of ML models to screen the status and type of diabetes trained on publicly available gut microbiome data from the American Gut Project for both classifications. We tested the top 1000, 500 and 100 most variable sOTUs across the samples to train the DT, EN, RF and SVMRK classifiers on 70% of the original features dataset. While the AUC scores obtained for the RF and DT classifiers were robust, they displayed low sensitivity, indicating that a significant proportion of true positives were misclassified as negatives. Increasing the maximum number of iterations in the models and the threshold for discrete classification produced similarly robust AUC values but models with both higher sensitivity and specificity.

The initial diabetes status screening model batch performed on the 1000 most variable features achieved AUC scores of around 0.7, but sensitivities lower than 50%, which indicates that models using 1000 features could only classify one-half of the positive cases. Additionally, the high specificity displayed in these models might not indicate a good performance when classifying negatives, but rather a bias towards negative classification, which would entail a high number of false negatives. This poor performance suggests that the bigger dataset used for model training produced overfitted models. The reduction of the dataset to 500 and 100 features produced better-fitted models after modulating the classification threshold and iterations maxit. In this case, reducing the dataset size by eliminating irrelevant or redundant features may have led to better-fitted ML models by mitigating overfitting, reducing multicollinearity and mitigating data sparsity. Sensitivity increased by 74% from 0.46 to 0.80 for the RF models of status, while AUC increased from 0.76 to 0.77 after the dataset was reduced to 500 features.

Other studies have used similar approaches to classify diabetes. One study that combined neural network and RF identified T2DM microbial markers using species-level taxonomic features and gene-level profiles of subjects [41]. A similar study applied an interpretable ML framework to design a microbiome risk score of T2DM, which was confirmed *in vivo* by faecal transplantation in germ-free mice [42]. While using taxonomic features to train ML algorithms is common [43,44], we used high-variance sOTU features instead of enriched taxa, as operational taxonomic units may represent finer and more specific levels of microbial diversity than taxonomic features [34]. The use of variable high-variance sOTU features allows to reduce the dimensionality of the training dataset and to improve model performance in the testing stage [34]. In this study, first, we normalized frequencies in percentages and then used log10 normalization to reduce skewness and improve interpretability. Previous studies have also applied ML models to datasets without normalization to assess the classifying capacity of raw data and to avoid repeated pre-processing [22]. However, ML frameworks from large genomic datasets have the downside of introducing batch [45] and bloom effects [46]. More complex normalization strategies, such as the supervised normalization used by Xu *et al.* [43], will be needed to reduce heteroscedasticity when implementing ML frameworks using high-dimensional genomic data produced through numerous batches and pipelines to apply them to real-life disease prediction scenarios.

Significant changes between the gut microbiota composition of diabetes and non-diabetes subjects were observed. The same was observed for diabetes types. In this study, increased levels of *Bacteroides* and *Prevotella* were found in positive-status subjects, while decreased levels of *Faecalibacterium* and *Pseudomonas* were displayed in the negative-status group. Patients with T2DM also presented a lower abundance of *Faecalibacterium* as well as fewer *Bacteroides*. *R. torques* and *Blautia* were enriched in the positive-status group, while *R. faecis* and *B. obeum* were enriched in T1DM subjects. *R. faecis* also marked the negative-status group. *Roseburia* and *Blautia* were also among the most informational features in the models. Increased abundance of *B. obeum* and *Blautia wexlerae* has been positively correlated with insulin resistance in pre-diabetes subjects [47]. *R. torques* was found enriched in mice colonized with obese phenotype microbiome cultures, where they were correlated with increased butyrate, acetate, 3,4 dihydroxyphenyl acetate, pyroglutamate and xylose [18]. However, in the same study, *B. obeum* was enriched in mice colonized by lean phenotype microbiota, and different *Clostridium* strains and species were enriched in both groups [18]. *Lachnospira*, found here enriched in the negative-status group, has been found to be markers of the control group and acts as a probiotic when compared with T2DM groups [48]. Zhang *et al.* found that *Lachnospira*, along with other short-chain fatty acid (SCFA)-producing bacteria, such as *Roseburia* and *Clostridium*, are significantly reduced in T2DM subjects [48]. *Lachnospira* and certain *Clostridiaceae* features were markers of healthy subjects in this study, pointing at their commensal function in the gut microbiome. A decrease in the abundance of Gram-positive SCFA-producing bacteria and an increase in Gram-negative endotoxin-producing bacteria is characteristic of subjects with T2DM [49]. *Roseburia* has been found to be negatively correlated with obesity and T2DM, and due to their role in the modulation of glucose homoeostasis, its dysbiosis has been proposed to play a role in the progression from normoglycaemia to prediabetes [50]. While *Roseburia* sOTU was found enriched in the positive-status subjects, which has been reported before for diabetic groups [51], *R. faecis* was a microbial marker of the negative-status group. Another of the high-contributing sOTUs across all models was *F. prausnitzii*, which was enriched in the negative-status group. Similarly, the abundance of *Faecalibacterium* was reduced in diabetes patients in various studies when compared with healthy controls [52,53]. This aligns with studies showing a reduction in the abundance of *Clostridiales*, particularly in *Roseburia* species and *F. prausnitzii*, in patients with T2DM [54]. In addition to this, different members of several taxa, like *Bacteroides*, *Prevotella*, *Ruminococcus* or *Clostridium*, that by themselves are among the most populous microbes of the human gut [54], may be linked to differing conditions, such as the case of different *Bacteroides* taxa found here and in other works [18].

This known link between the gut microbiota and diabetes status presents a promising avenue for disease prediction [51], which necessitates the application of systems biology and a more in-depth understanding of the mechanistic relations between the dysbiotic microbiota and the body [55]. Previous studies have implemented ML frameworks to reveal strong connections between diabetes and microbial profiles of the gut [42] and to predict diabetes and diabetes complications like diabetic retinopathy using different biotic and abiotic variables [56]. It is important to consider that variability in the microbiome composition among healthy individuals also challenges the applicability of the microbiome as a screening tool [57]. Additionally, changes in the physiology of diabetics and prediabetics, as well as medication intake, have the potential to alter the gut microflora, which needs to be considered when implementing approaches like these [58].

While LEfSe classified markers for diabetes types, our models provided poor metrics for diabetes typing. One of the main limitations of the present study was the unbalanced nature of the dataset used for the typing of diabetes, which may have contributed to the low sensitivity of this test. Future studies, particularly those conducted in controlled clinical settings, should prioritize more balanced datasets. This would help mitigate potential biases, improve model sensitivity and offer more reliable predictions for diabetes typing. Additionally, external validation using independent metagenomic datasets, which was not performed here, would enhance the generalizability of the ML models and ensure their robustness and applicability across diverse populations and settings. Another limitation is the random sampling approach used in this study, which did not control for specific demographic factors. While this approach aimed to reflect real-world diversity, it may allow underlying demographic biases from the original dataset to persist. Overall, future studies will benefit from increased sample sizes, better-balanced datasets [59] and the inclusion of other widely used patient variable features as predictors [42]. Despite these limitations, this study is an exploratory step for the use of gut microbiome features for diabetes classification and for the construction of robust and clinically applicable predictive models.

Here, we used high-variance sOTU features to train ML models for classification of diabetes status. This study demonstrates that high-variance metagenomic features derived from gut microbiome data can effectively train ML models for diabetes classification. Prioritizing feature selection to reduce dataset dimensionality helps mitigate overfitting and enhances model performance. Ensuring dataset balance and external validation with independent datasets, as well as the inclusion of demographic and clinical variables, can lead to improved model generalizability and clinical applicability. Overall, there is potential for integrating ML and microbiome profiling for early disease prediction and precision medicine, an approach that can also be explored in multifactorial diseases with no apparent causative link with the microbiome composition.

## supplementary material

10.1099/mgen.0.001365 Uncited Supplementary Material 1.

10.1099/mgen.0.001365 Uncited Supplementary Material 2.

## Abbreviations

AUC: area under the receiver operating characteristic curve
DT: decision tree
IBD: inflammatory bowel disease
LDA: linear discriminant analysis
LEfSe: linear discriminant analysis effect size
ML: machine learning
PCoA: principal coordinate analysis
PERMANOVA: permutational multivariate analysis of variance
PPV: positive predictive value
RF: random forest
SCFA: short-chain fatty acid
sOTUs: sub-operational taxonomic units
SVMRK: support vector machine with radial kernel
T1DM: type 1 diabetes mellitus
T2DM: type 2 diabetes mellitus
