# Supplementary material for: Using gut microbiome metagenomic hypervariable features for diabetes screening and typing through supervised machine learning
Source: Microb Genom. 2025 Mar 10;11(3):001365. doi: 10.1099/mgen.0.001365 (PMC11893737; doi:10.1099/mgen.0.001365)
Supplement: Uncited Supplementary Material 1. [file mgen-11-01365-s001.pdf]

Supplementary Materials for

Using gut microbiome metagenomic hypervariable features for diabetes screening and typing through supervised machine learning

Xavier Chavarria[1,*], Hyun Seo Park[1,2], Singeun Oh[1], Dongjun Kang[1], Jun Ho Choi[1], Myungjun Kim[1], Yoon Hee Cho[1], Myung-hee Yi[1], and Ju Yeong Kim[1,*]

[1] Department of Tropical Medicine, Institute of Tropical Medicine, and Arthropods of Medical Importance Resource Bank, Yonsei University College of Medicine, Yonsei-ro 50-1, Seodaemun-gu, Seoul 03722, Republic of Korea

[2] Department of Systems Biology, Yonsei University College of Life Science and Biotechnology, Yonsei-ro 50-1, Seodaemun-gu, Seoul 03722, Republic of Korea

*Corresponding author: E-mail: jykim0802@yuhs.ac

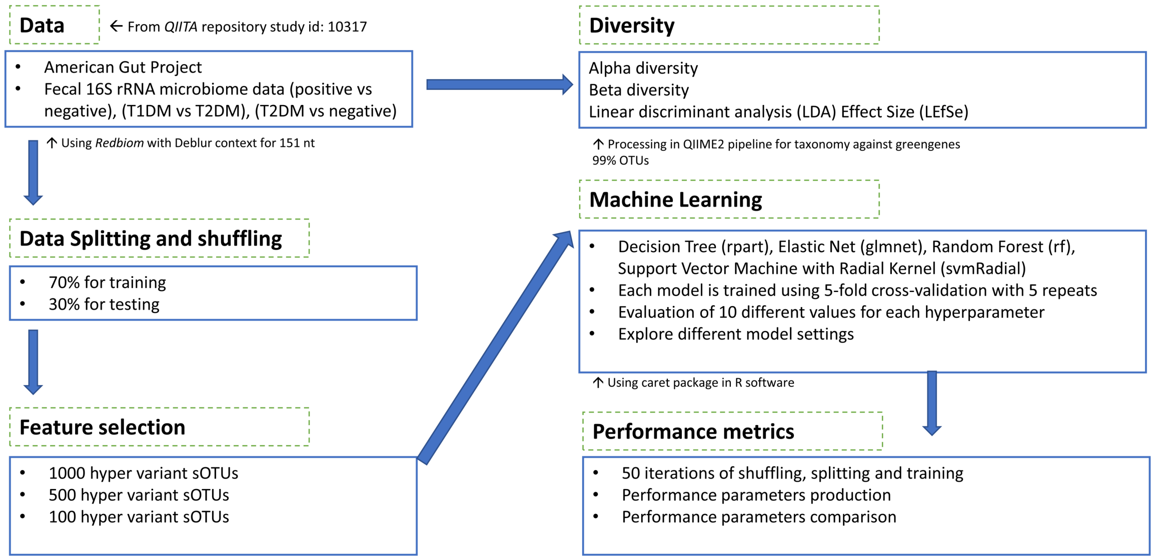

Supplementary Figure S1. Methodology workflow of the present study.

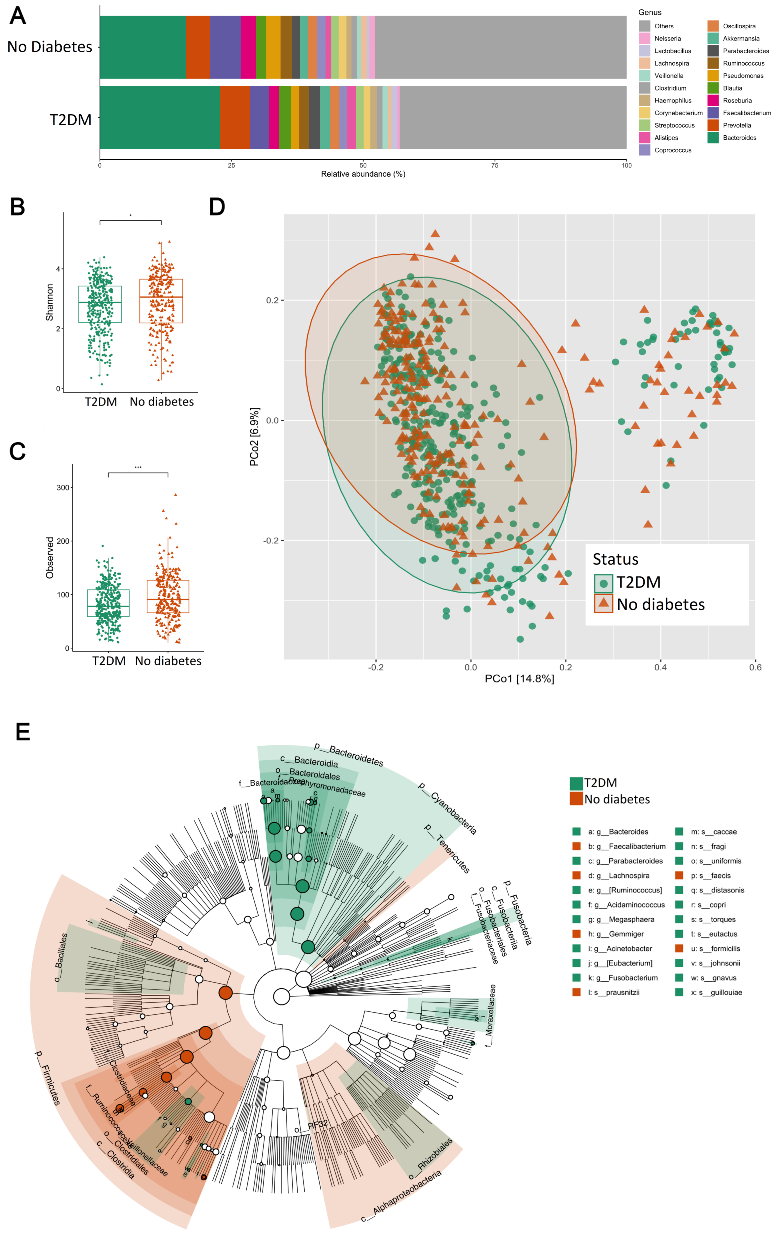

Supplementary Figure S2. Diversity metrics of the gut microbiome from volunteers that reported T2DM vs. volunteers that did not report diabetes. Mean relative abundance of the top 20 gut microbiome sOTUs of volunteers that reported T2DM vs. volunteers that did not report diabetes at the genus level (A). Shannon index of the gut microbiome sOTUs of volunteers that reported T2DM vs. volunteers that did not report diabetes compared by Wilcoxon rank-sum test (B). Richness of the gut microbiome sOTUs of volunteers that reported diabetes vs. volunteers that did not report diabetes compared by Wilcoxon rank-sum test (C). Unweighted UniFrac distances PCoA of the gut microbiome of volunteers that reported diabetes vs. volunteers that did not reported diabetes tested with PERMANOVA (D). Cladogram of the top 50 more representative taxa based on LEfSe analysis from the gut microbiome of volunteers that reported diabetes vs. volunteers that did not report diabetes showing their phylogenetic relationship with the top 1000 most abundant taxa, labels in the cladogram show sOTUs (LDA > 2, p < 0.05) of superior taxonomic levels, labels outside the cladogram represent enriched sOTUs at the genus and species level (E).

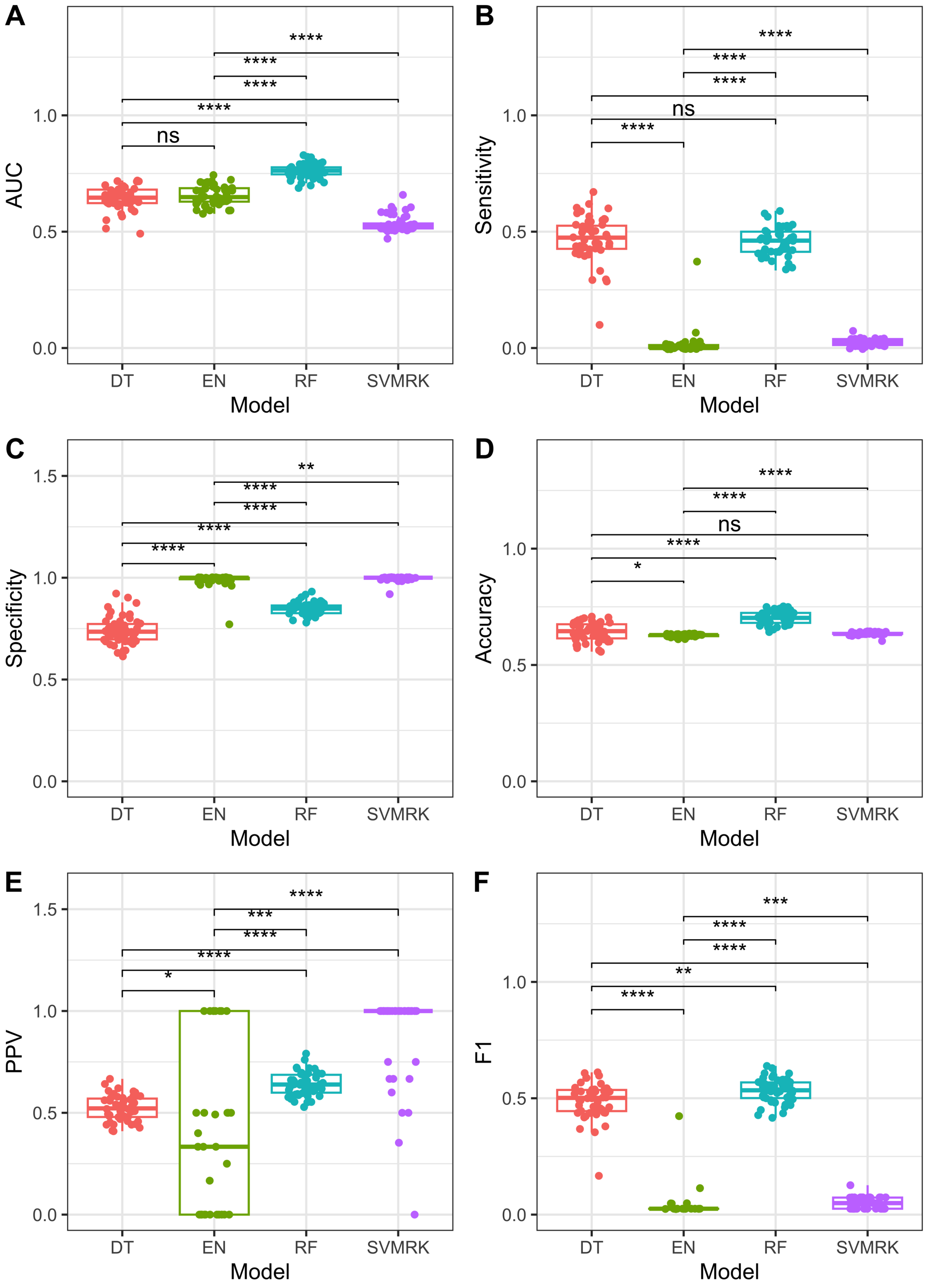

Supplementary Figure S3. Performance metrics of four supervised machine learning algorithms for the screening of diabetes trained on the top 1000 hyper-variable OTUs of the gut microbiome across 50 iterations. Diabetes status was screened with the Decision Tree (DT), Elastic Net (EN), Random Forest (RF), and Support Vector Machine with Radial Kernel (SVMRK) models. Area under the receiver operating characteristic curve (A), sensitivity (B), specificity (C), accuracy (D), Positive predictive value (E), F-score (F). The models use a predicted probability classification threshold of 0.5 with default iterations and tree maxit levels.

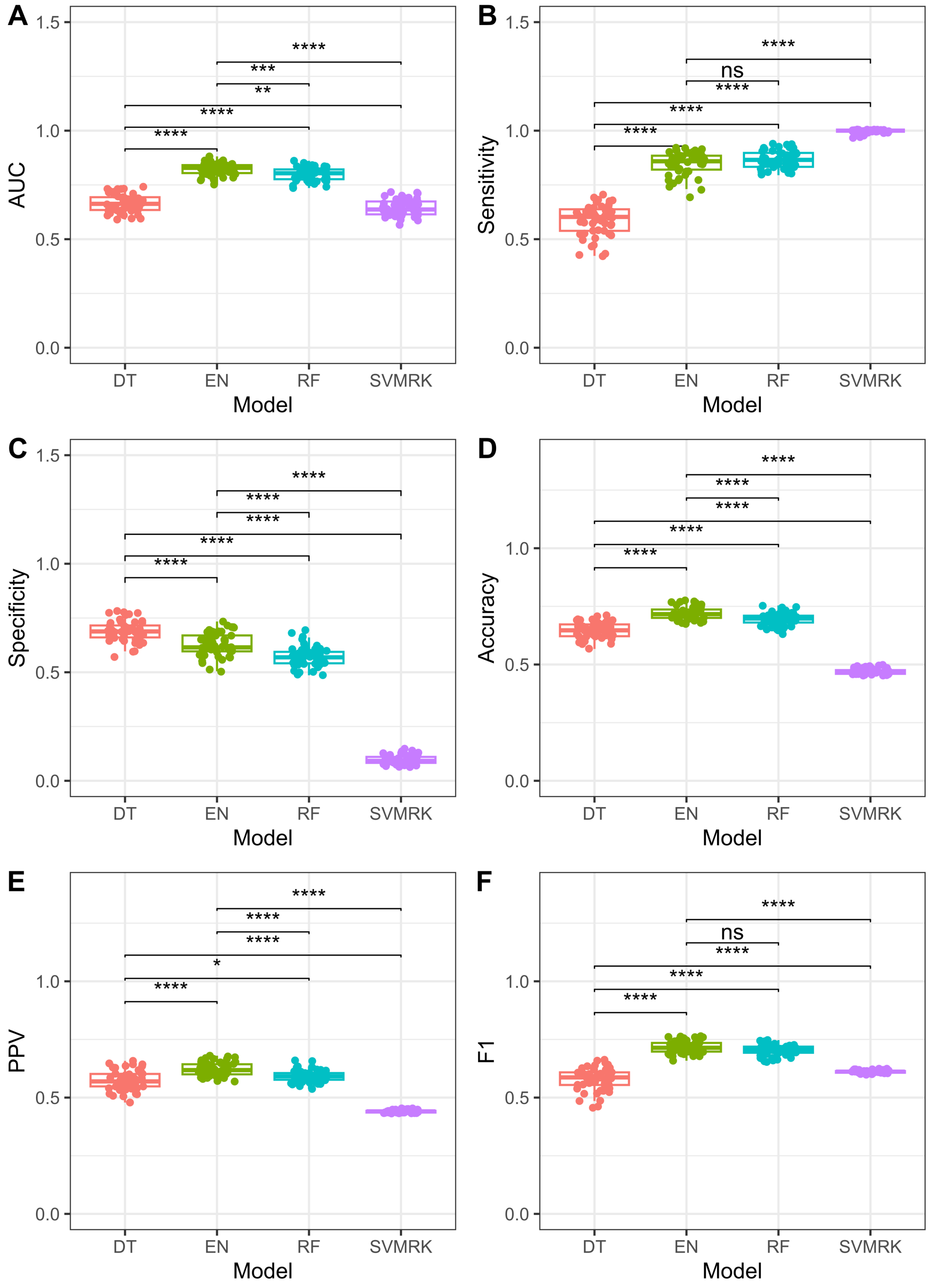

Supplementary Figure S4. Performance metrics of four supervised machine learning algorithms for the screening of diabetes classifying negative status vs T2DM trained on the top 500 hyper-variable OTUs of the gut microbiome across 50 iterations. Diabetes status was screened with the Decision Tree (DT), Elastic Net (EN), Random Forest (RF), and Support Vector Machine with Radial Kernel (SVMRK) models. Area under the receiver operating characteristic curve (A), sensitivity (B), specificity (C), accuracy (D), Positive predictive value (E), F-score (F). The models use a predicted probability classification threshold of 0.65 with 10000 maximum iterations for the Elastic Net (EN) and Support Vector Machine with Radial Kernel (SVMRK) models, maximal depth of 30 for the Decision Tree (DT), 1000 maximum trees for the Random Forest (RF) models.
